# Unusual diagnosis of feline cardiac lymphoma using cardiac needle biopsy

**DOI:** 10.1186/s12917-022-03357-7

**Published:** 2022-06-28

**Authors:** S. Tanaka, R. Suzuki, M. Hirata, Y. Kagawa, H. Koyama

**Affiliations:** 1 Alpha Animal Hospital, 1075-1 Imai, Kawanakajima, Nagano, 381-2226 Japan; 2 Nippon Veterinary and Life Science University, 1-7-1 Kyonan-cho, Musashino, Tokyo 180-8602 Japan; 3 Japan Small Animal Medical Center, 1-10-4 Wada, Higashi-Tokorozawa, Tokorozawa, Saitama, 359-0023 Japan; 4 IDEXX JAPAN, 3-5-8 Saiwai-cho, Fuchu, Tokyo 183-8509 Japan; 5 North Lab, 8-35 Kita, Hondori 2-chome, Shiraisi-ku, Sapporo, Hokkaido 003-0027 Japan

**Keywords:** Feline lymphoma, Two-dimensional speckle-tracking echocardiography, Cardiac troponin I, Myocardial hypertrophy, Cardiac tumors

## Abstract

**Background:**

Cardiac tumors in cats are relatively rare, with lymphoma accounting for more than half of all cases. However, feline cardiac lymphoma is often diagnosed post-mortem, and it is difficult to diagnose while the cat is still alive. It is the first report of a direct, rather than estimative, diagnosis with cardiac needle biopsy of a living cat with cardiac lymphoma.

**Case presentation:**

A 3-year-old domestic short-haired male cat experienced loss of energy and loss of appetite. Thoracic radiography and transthoracic echocardiography showed cardiomegaly with slight pleural effusion and cardiac tamponade due to pericardial effusion, respectively. In addition, partial hyperechoic and hypertrophy of the papillary muscle and myocardium were observed. Blood test showed an increase in cardiac troponin I levels. Pericardial fluid, removed by pericardiocentesis, was analyzed; however, the cause could not be determined. With the owner’s consent, pericardiectomy performed under thoracotomy revealed a discolored myocardium. Cardiac needle biopsy was performed with a 25G needle, and a large number of large atypical lymphocytes were collected; therefore, a direct diagnosis of cardiac lymphoma was made. Pathological examination of the pericardium diagnosed at a later date revealed T-cell large cell lymphoma. The cat underwent chemotherapy followed by temporary remission but died 60 days after the diagnosis. Postmortem, two-dimensional speckle-tracking echocardiography (data when alive) revealed an abnormal left ventricular myocardial deformation, which corresponded to the site of cardiac needle biopsy.

**Conclusions:**

This rare case demonstrates that cardiac lymphoma should be added to the differential diagnosis in cats with myocardial hypertrophy and that the diagnosis can be made directly by thoracotomy and cardiac needle biopsy. In addition, the measurement of cardiac troponin I levels and local deformation analysis of the myocardium by two-dimensional speckle-tracking echocardiography may be useful in the diagnosis of cardiac tumors.

**Supplementary Information:**

The online version contains supplementary material available at 10.1186/s12917-022-03357-7.

## Background

Cardiac tumors in cats are relatively rare, with lymphoma accounting for more than half of all cases. However, feline cardiac lymphoma is often diagnosed post-mortem [1–12], and it is difficult to diagnose while the cat is still alive. It is the first report of a direct, rather than estimative, diagnosis with cardiac needle biopsy of a living cat with cardiac lymphoma.

## Case presentation

A 3-year-old domestic short-haired male cat experienced loss of energy and loss of appetite. Physical examination at the initial visit showed a body temperature of 38.0 °C, normal auscultation, normal visible mucosa, and slightly elevated blood pressure (systolic blood pressure: 165 mmHg). The cat also had right-sided Horner's syndrome of undetermined etiology, as no gross pathology was found in the right ear canal or neck. Blood chemistry showed elevated levels of plasma total protein and abnormally high levels of cardiac troponin I (cTnI) (1.18 ng/mL; reference range, 0–0.17 ng/mL: i-STAT Heska [13]).

Electrocardiogram findings revealed sinus tachycardia (253 bpm), and thoracic radiography showed no abnormalities. Echocardiography revealed irregular hypertrophy of the anterior papillary muscle and increased echogenicity; however, there were no evidence of congestion such as enlargement of the left atrium (Video 1). A clinical diagnosis of suspected hypertrophic cardiomyopathy (HCM) phenotype (include myocarditis) was made, and the cat was observed on follow-up.

On the 12th day from the initial visit, the cat was brought to the hospital again with the chief complaint of syncope. At the time of admission, the cat presented with open-mouth panting, but no signs of cyanosis were observed. Blood tests showed a higher cTnI level (3.25 ng/mL), and a complete blood count analysis showed eosinophilia. The cat tested positive for both feline immunodeficiency virus and feline leukemia.

Thoracic radiography revealed cardiomegaly (VHS = 8.5; reference range, 7.5 ± 0.3) with slight pleural effusion. Focused cardiac ultrasound revealed pericardial effusion and increased hypertrophy of the papillary muscles and left ventricular posterior wall. The patient also had right atrial collapse, which was diagnosed as cardiac tamponade (Table 2, Video 2, 3), and 10 mL of pericardial fluid was removed.

The total protein of the pericardial fluid was 5.2 g/dL, the number of nucleated cells was 8300 /mL, and a mixture of neutrophils; macrophages; and large, medium, and small lymphocytes were observed in the sediment smear. These findings are consistent with chronic active inflammation.

**Fig. 1:**
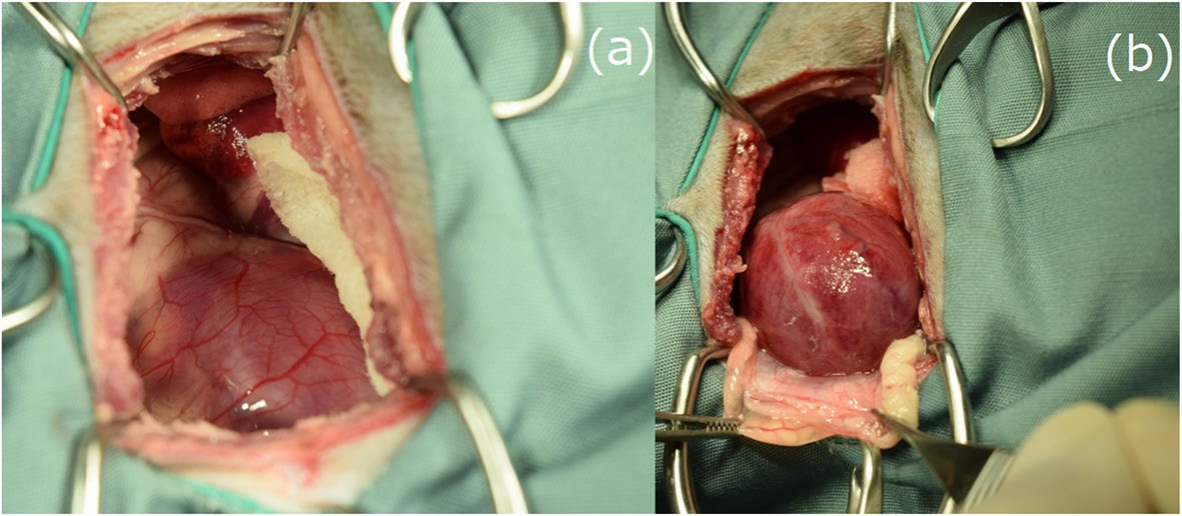
Before pericardial removal. **a** After pericardium removal. **b** The pericardium is slightly thickened and has abundant blood vessels, and the myocardium on the free wall side of the left ventricle after removal of the pericardium is reddish and has a rough surface

**Fig. 2:**
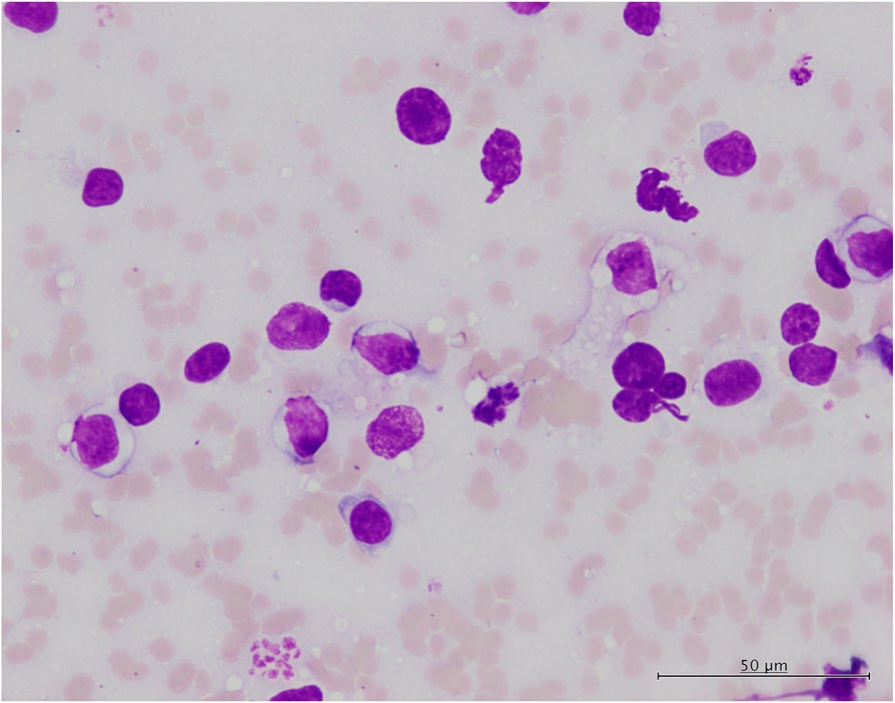
Cytology from myocardial needle biopsy. The image shows many lymphoid cells with large dysmorphic nuclei. (Captured by Olympus BX-43 and DP-27, the resolution is 150 dpi, uncompressed)

After consultation with the owner, pericardiectomy was performed through thoracotomy. The pericardium was slightly thickened and had abundant blood vessels (Fig. 1-a). The left ventricular free wall myocardium had a reddish and rough surface that was found after pericardial removal (Fig. 1-b). The free wall myocardial surface was punctured six to seven times with a 25-G needle without syringe aspiration. There was minimal bleeding from this procedure, and hemostasis was achieved by adding pressure with a cotton swab. No arrhythmia was observed during the procedure. No problems with this procedure occurred after the operation. Cardiac needle biopsy cytology revealed many large, atypical lymphocytes (Fig. 2), which led to a direct diagnosis of cardiac lymphoma on the 12^th^ day from the initial visit.

**Fig. 3:**
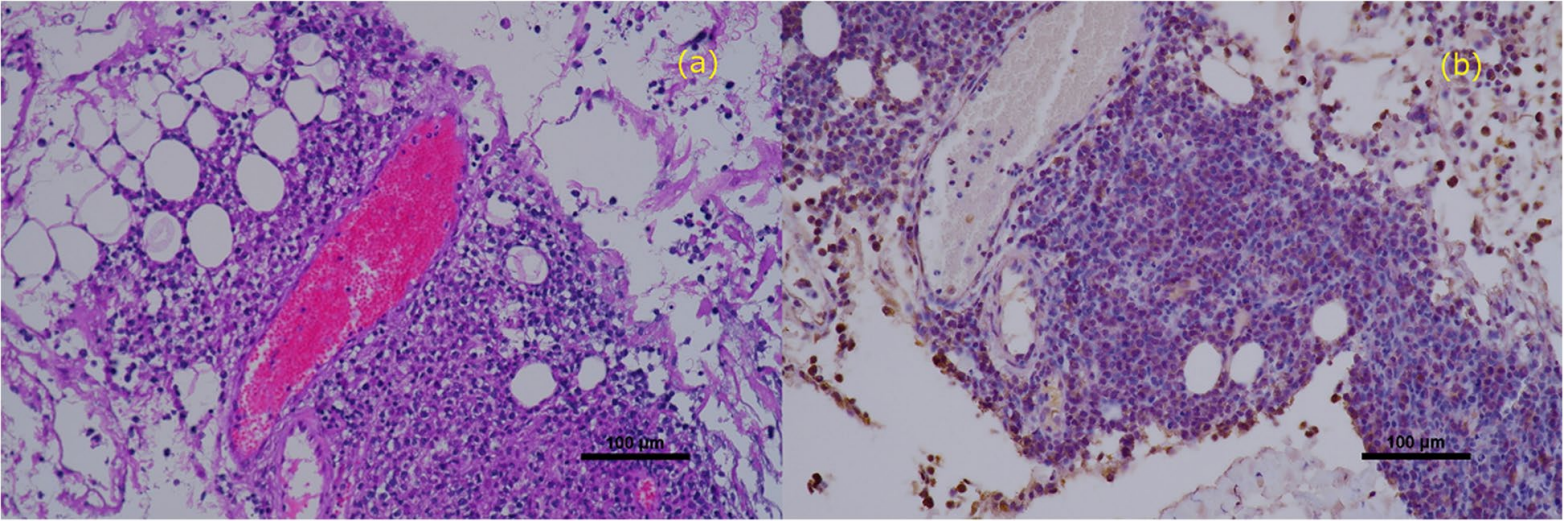
Pericardial pathology. Immunostaining image showing CD3-positive/CD20-negative lymphocytes, indicating a diagnosis of a T-cell large cell lymphoma. Hematoxylin and eosin staining (× 20) (**a**) CD3 immunostaining (× 20) (**b**)

**Fig. 4:**
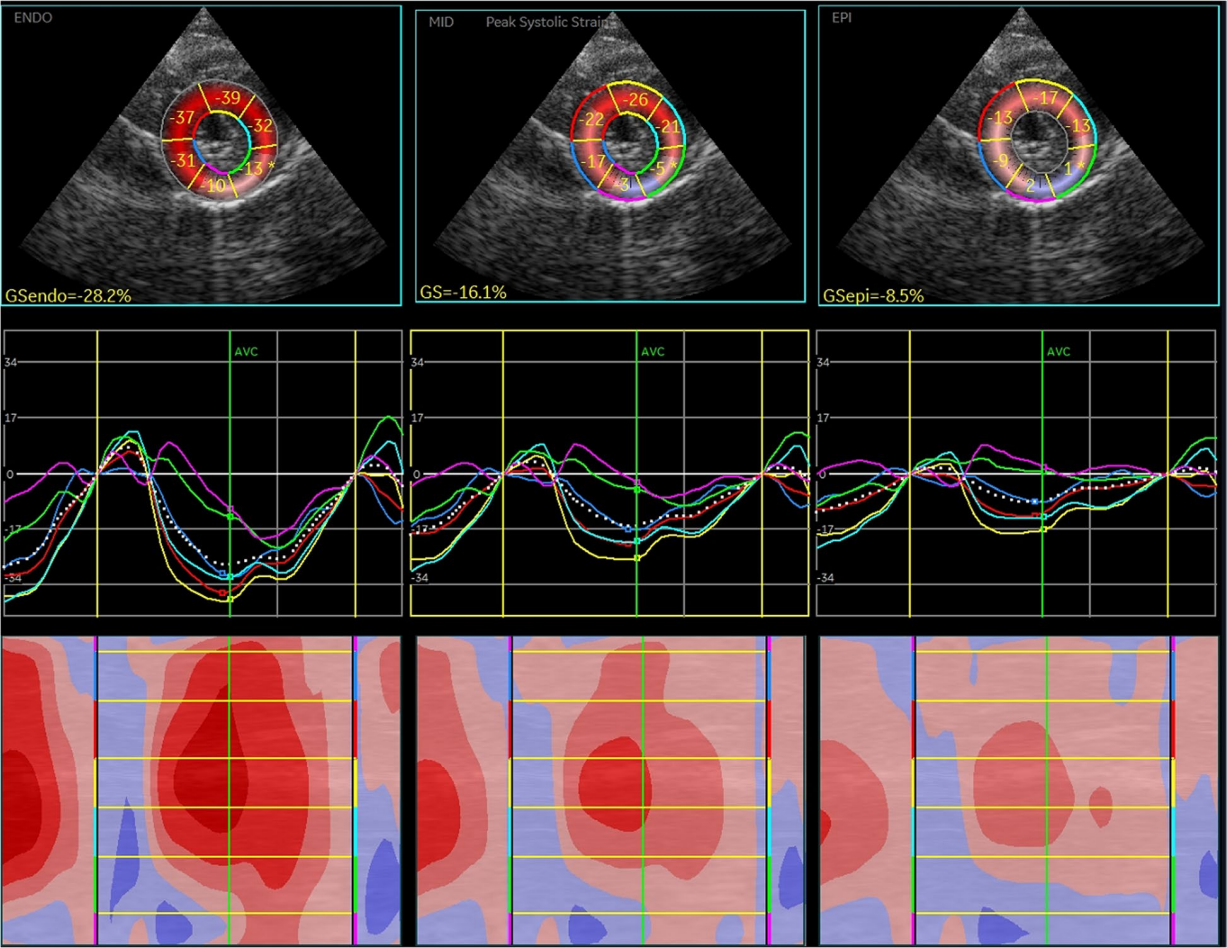
Two-dimensional speckle-tracking echocardiography (on the 4th day after diagnosis). The green lines indicate myocardial deformations of the left ventricular free wall. This site corresponds to the site of myocardial hypertrophy and cardiac needle biopsy. From left to right: results for the endomyocardial layer, whole layer of the myocardium, and the epimyocardial layer. Note the significant decrease in green (segmental strain value is -5%) and pink myocardial site (segmental strain value -3%) in whole layer strain (center)

One day after the diagnosis, the cat received an injection of L-asparaginase (400 IU/kg/subcutaneous injection [SC]), followed by chemotherapy. Subsequently, the following chemotherapy protocol was adhered to: vincristine 0.5 mg/m^2^/intravenous injection (IV) on the 8th, 22nd, and 36th days after diagnosis; cyclophosphamide 10 mg/kg/IV on the 15th day after diagnosis; methotrexate 0.8 mg/kg/IV on the 29th day after diagnosis; L-Asparaginase 400 IU/kg/SC again on the 43rd day after diagnosis; doxorubicin 1 mg/kg/IV on the 50th day after diagnosis; and cytarabine 100 mg/m^2^/SC on the 57th and 58th days after diagnosis. We received the results of the pericardial pathological examination six days after the clinical diagnosis. Immunostaining revealed CD3 positive/CD20 negative lymphocytes, and T-cell large cell lymphoma was diagnosed (Fig. 3). On the 15th day after diagnosis, cTnI levels returned to normal (Table 1), and myocardial morphology appeared to have normalized (Table 2, Video 4). On the 43rd day after diagnosis, relapse was observed in places other than the heart. The cat died due to neurological symptoms on the 60th day after diagnosis. The owner did not consent for autopsy. Before death, a mild increase in cTnI levels and papillary muscle hypertrophy were noted, a third papillary muscle was more evident (Video 5), but there were no signs of left atrial enlargement or heart failure (Table 2). Postmortem, two-dimensional speckle-tracking echocardiography (2D-STE) analysis was performed using the echocardiographic data before death (4^th^ day after diagnosis). The cardiac needle biopsy site (myocardium near the anterior papillary muscle of the left ventricle) revealed a marked reduction in myocardial deformity and lack of coordination with other areas of the left ventricular myocardium (Fig. 4, green line).

**Table 1:** Chronological presentation of cardiac troponin I levels and chemotherapy regimen

Day	cTnI (ng/mL)	Chemotherapy regimen
1	1.18	-
12 (Diagnosis date)	3.25	-
13	-	L-Asp 400 IU/kg/SC
14	1.24	-
16	0.60	-
20	0.17	VCR 0.5 mg/m^2^/IV
27	0.07	CPM 10 mg/kg/IV
34	0.05	VCR 0.5 mg/m^2^/IV
41	0.03	Met 0.8 mg/kg/IV
48	0.02	VCR 0.5 mg/m^2^/IV
55	0.04	L-Asp 400 IU/kg/SC
62	0.06	Dox 1 mg/kg/IV
69	0.17	Ara-C 100 mg/m^2^/SC
70	-	Ara-C 100 mg/m^2^/SC

*Ara-C* Cytarabine, *CPM* Cyclophosphamide, *Dox* Doxorubicin, *IV* Intravenous injection, *L-Asp* L-asparaginase, *MET* Methotrexate, *SC* Subcutaneous injection, *VCR* vincristine

**Table 2:** Results of echocardiography

Day	1 (Video 1)	12 (Video 2,3)	27 (Video 4)	69 (Video 5)
IVSd (mm)	3.8	4.0	4.0	3.3
LVPWd (mm)	3.1	5.5	4.3	3.4
LVIDd	15.4	14.1	13.8	14.4
LA/Ao	1.2	1.2	1.4	1.3
APM (mm)	8.2	12.0	7.7	8.0
TPM (mm)	8.3	13.2	5.3	6.1

*IVSd* Interventricular septum diameter during diastole, *LVPWd* Left ventricular posterior wall diameter during diastole, *LVIDd* Left ventricular internal dimension during diastole, *LA/Ao* Left atrial/ aortic diameter ratio, *APM* Anterior papillary muscle, *TPM* Third papillary muscle

## Discussion and conclusions

Cardiac tumors in cats are relatively rare, with lymphoma accounting for more than half of all cases [1–12]. However, feline cardiac lymphoma is often diagnosed post-mortem, and it is difficult to diagnose while the cat is still alive. Delayed diagnosis may lead to delayed treatment, to the cat’s detrimental condition. In previous reports, most cases were clinically diagnosed as "estimated cardiac lymphoma" by analysis of stored pericardial fluid [3, 4, 8] or as lymphoma spillover from other sites and based on the patient’s response to chemotherapy [1, 9]. This is the first report of direct diagnosis with cardiac needle biopsy and early treatment of a living cat with cardiac lymphoma. In addition, as demonstrated in this case, cardiac troponin measurement and 2D-STE analysis may be useful diagnostic tools.

Although there is literature evaluating the safety of cardiac needle biopsy in dogs, there are no reports on this aspect in cats [14].

However, based on the abnormally high level of troponin in this case, which was different from the typical HCM findings, aggressive tests such as cardiac needle biopsy were performed. In this case, open thoracic pericardiectomy was performed for treating cardiac tamponade, during which we performed a cardiac needle biopsy under visualization. Although there are no reports that have evaluated safety in cats, it is likely that puncture with visualization can be performed safely by avoiding major coronary arteries.

In this case, HCM phenotype was suspected and clinically diagnosed because of hypertrophy of the left ventricular papillary muscle at the initial visit. In the ACVIM consensus statement, feline myocardial disease is classified by a clinical morphological approach; accordingly, a diagnosis of the HCM phenotype was made in the present case [15]. However, as opposed to traditional cases of HCM, troponin I values were markedly higher [16]. Furthermore, 2D-STE analysis of the left ventricular myocardium revealed a regional decrease in myocardial function coincident with the hypertrophic and biopsy area; these regional abnormalities are in contrast to typical myocardial function in HCM [17]. In addition to HCM, although they are rare, neoplastic diseases such as lymphoma, myocarditis, and toxoplasma infection have also been reported as myocardial diseases for differentiating hypertrophic myocardium in cats. In such myocardial diseases, cardiac needle biopsy cytology may be useful for diagnosis [18–20]. As in this case, cardiac needle biopsy cytology may be a more aggressive diagnostic test in cats with unexplained myocardial hypertrophy and that present with clinical findings different from HCM, especially in cats with abnormally high cTnI levels and abnormal myocardial function based on 2D-STE analysis. Furthermore, the abnormally high cTnI values in this case allowed us to focus on the heart after the cat’s initial visit.

cTnI has already proven useful in numerous studies, as it is an indicator of myocardial damage [16, 21]. In this case, the cTnI values decreased with the start of chemotherapy and normalized two weeks later. They increased again on the 50th day after diagnosis. According to recent studies, there is a correlation between myocardial damage and cTnI values in mice and rats [22]. Therefore, troponin levels may be a diagnostic reflection of myocardial damage due to tumor cell infiltration into the tissue. In this case, 2D-STE analysis showed local abnormalities in myocardial function. Although 2D-STE analysis has been used in past studies for the clinical diagnosis and differentiation of cats with cardiomyopathy [17, 23], it is often due to motor dysfunction of the entire left ventricle rather than a focal myocardial abnormality. Interestingly, in this case, local myocardial dysfunction was observed at the papillary muscle attachment site. However, pathological examination did not reveal tumor cell infiltration distribution or the extent of other myocardial damage such as myocardial necrosis. We believe that, in future cases, 2D-STE will be a useful tool in the diagnosis of cardiac tumors.

In this case, cardiac lymphoma was directly diagnosed by performing thoracotomy and cardiac needle biopsy cytology. Based on the results of these diagnostics, immediate intervention began with chemotherapy. In cats with suspected cardiac tumors associated with cardiac tamponade, cardiac needle biopsy cytology, in addition to thoracotomy pericardial resection, may be a useful tool for early diagnosis and therapeutic intervention. In addition, measurement of cTnI levels and detection of myocardial damage using 2D-STE may also be useful as a diagnostic aid in these cases.

## Supplementary Information

**Additional file 1: Video 1.** At initial presentation. (Right parasternal short axis view of the papillary muscle level). The video shows the irregular hypertrophy of the anterior papillary muscle and increased echogenicity.

**Additional file 2: Video 2.** On day of the diagnosis. (Right parasternal short axis view of the papillary muscle level). The video shows pericardial effusion and further hypertrophy of the papillary muscles and left ventricular posterior wall.

**Additional file 3: Video 3.** On day of the diagnosis. (Right parasternal long axis view). The video shows cardiac tamponade (right atrial collapse).

**Additional file 4: Video 4.** On the 15th day after diagnosis. (Right parasternal short axis view of the papillary muscle level). The video shows that myocardial morphology appeared to have normalized.

**Additional file 5: Video 5.** On the 57th day after diagnosis. (Right parasternal short axis view of the papillary muscle level). The video shows that papillary muscle hypertrophy has returned.

## Data Availability

The data that support our findings are available from the corresponding author on reasonable request.

## Abbreviations

cTnI: Cardiac troponin I
HCM: Hypertrophic cardiomyopathy
2D-STE: Two-dimensional speckle-tracking echocardiography
